# A Novel Defensin-Like Peptide Associated with Two Other New Cationic Antimicrobial Peptides in Transcriptome of the Iranian Scorpion Venom

**DOI:** 10.18869/acadpub.ibj.21.3.190

**Published:** 2017-05

**Authors:** Masoumeh Baradaran, Amir Jalali, Maryam Naderi soorki, Hamid Galehdari

**Affiliations:** 1 Toxicology Research Center, Ahvaz Jundishapur University of Medical Sciences, Ahvaz, Iran; 2 Department of Pharmacology and Toxicology, Toxicology Research Center, Ahvaz Jundishapur University of Medical Sciences, Ahvaz, Iran; 3 Department of Genetics, Sciences Faculty, Shahid Chamran University, Ahvaz, Iran

**Keywords:** Antimicrobial cationic peptides, Defensin-like peptide, *Mesobuthus eupeus*

## Abstract

**Introduction:**

Scorpion venom is a source of bioactive peptides, and some antimicrobial peptides (AMPs) have been found in the venom gland of scorpions. Therefore, the discovery of new anti-infective agents is an essential need to overcome the problem of antibiotic resistance of clinical isolates. Here, we describe three new cationic AMPs, including meuVAP-6, meuAP-18-1, and meuPep34 from the venom gland of the Iranian scorpion, *Mesobuthus eupeus*.

**Methods:**

The cDNA sequences encoding all the three peptides were obtained from the cDNA library of scorpion venom gland and were deposited in the GenBank database.

**Results:**

MeuVAP-6 and meuAP-18-1 are non-disulphide-bridged antimicrobial peptides, while meuPep34 is a cysteine-rich defensin-like peptide.

**Discussion:**

All three identified AMPs are rich in arginine and tryptophan. The overall results from the length, net charge, and hydrophobicity index suggested that meuPep34 could be the most active AMPs with the potential ability of biofilm inhibition. The data from molecular characterization of identified AMPs can provide a platform with application in drug discovery programs.

## INTRODUCTION

Defensins are a group of antimicrobial peptides (AMPs) and a part of innate immunity in vertebrates and invertebrates[1]. The first scorpion defensin was isolated from the hemolymph of the North African scorpion, *Leiurus quinquestriatus*, based on the similarity with insect defensin. It was also active against Gram-positive *M. luteus* but inactive against Gram-negative *E. coli*[2]. Later, some other AMPs with different effects were identified from various scorpion venomes[3].

AMPs as a part of the immunity system of all animals have the length of four to more than 100 amino acids and also are wide-spectrum molecules[4]. Finding new effective AMPs is valuable because resistance to available antibiotics is rising constantly, and the need for new antibiotics to replace is increasing[5,6]. One promising class of antibiotics is AMPS[7]. In the current study, we investigated the transcriptome of the Iranian scorpion *Mesobuthus eupeus* venom gland with the aim of finding new AMPs. This study represents the molecular and bioinformatics analysis of three founded AMPs.

## MATERIAS AND METHODS

Double-stranded cDNA was synthesized from the total RNA extracted from the venom glands of *M. eupeus*. Following the cDNA cloning in appropriate cloning vector, the recombinant vectors were transformed into the bacterial host to prepare the cDNA library. The cDNA library was then sequenced and analyzed to find the AMPs. Nucleotide BLAST and protein BLAST were carried out using online BLAST tool in NCBI (blast.ncbi.nlm.nih.gov), while open reading frames (ORFs) were detected using the online ORF finder tool (www.ncbi.nlm.nih.gov/orffinder/). Molecular weight, theoretical isoelectric pH, and the net charge of peptides (at neutral pH) were predicted using peptide property calculator online tool at Innovagen (www.innovagen.com/proteomics-too/ls). SignalP4.1 (http://www.cbs.dtu.dk/services/SignalP/) was also used to identify any signal peptide (http://www.cbs.dtu.dk/services/SignalP/). The hydro-phobicity of each peptide was calculated by GPMAW lite online tool available in http://www.alphalyse.com/gpmaw_lite.html. The secondary structure of peptides was predicted by using the GOR secondary structure prediction method software (npsa-prabi.ibcp.fr/cgi-bin/npsa_automat.pl? page=npsa_gor4.html)[8]. The alignment of every peptide was performed using Alignment tool in UniProt (www.uniprot.org/align). Transmembrane domains were predicted by the TMHMM tool (http://www.cbs.dtu.dk/services/TMHMM/). The schematic diagrams of protein domain structures were drawn by using the Illustrator for biological sequences (IBS, version 1.0) software (http://ibs.biocuckoo.org/).

## RESULTS

**Fig. 1:**
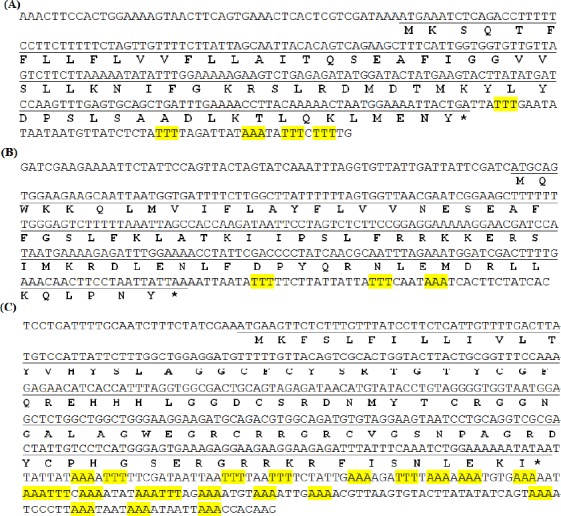
cDNA and translated open-reading frame (ORF)for meuVAP-6 (A), meuAP-18-1 (B), and meuPep34 (C). Nucleotides of ORF are single underlined, and stop codons are indicated by asterisks. AAA and TTT motifs in 3’UTR are highlighted with yellow color.

BLASTn analysis and protein BLAST identified four transcripts with acceptable similarity to the known scorpion AMPs (*E*<10^−3^). One of the transcripts namely CLAP has previously been identified and described by our team and deposited in NCBI with the accession number of KC108907[9]. Other transcripts were named meuVAP-6, meuAP-18-1, and meuPep34. As shown in Figure 1, the cDNA sequences of all the three peptides have been deposited in GenBank under the accession numbers KU513845 (meuVAP-6), KU513846 (meuAP-18-1), and KU513849 (meuPep34). The full length cDNA of meuVAP-6 is 314 bp that encodes a 70-amino acid peptide containing a 23-amino acid signal peptide, while that of meuAP-18-1 is 339 bp encoding a 76-amino acid peptide consisting of a 25-amino acid signal peptide. The full length cDNA of 508 bp encoding a 103-amino acid peptide, meuPep34, was also found, with a 20-amino acid predicted signal.

**Fig. 2:**
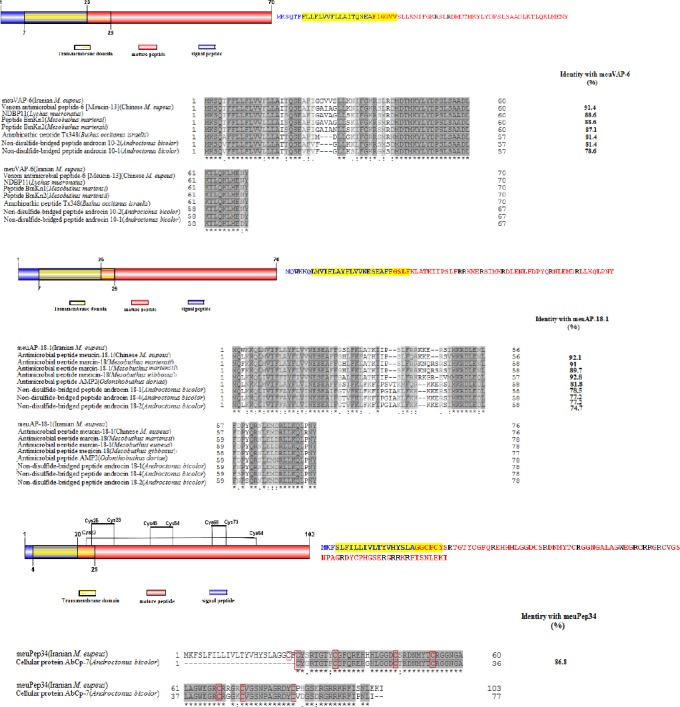
The left images are schematic image of (A) meuVAP-6, (B) meuAP-18-1, and (C) meuPep34 with its domains. The right images showes aa sequences of (A) meuVAP-6, (B) meuAP-18-1, (C) meuPep34 and aa of each domain. (A) Down image indicates aa sequences alignment of meuVAP-6 with homologues, including venom antimicrobial peptide-6 (Meucin-13), Chinese *M. eupeus* (AC. no.: E4VP07), NDBP11, *Lychas mucronatus* (AC. no.: A0A0U1S7U5), Peptide BmKn1, *Mesobuthus martensii* (AC. no.: Q9GQW4), Peptide BmKn2, *Mesobuthus martensii* (AC. no.: Q6JQN2), Amphipathic peptide Tx348, *Buthus occitanus israelis* (AC. no.: B8XH50), non-disulfide-bridged peptide androcin 10-2, *Androctonus bicolor* (AC. no.: A0A0K0LBS0), non-disulfide-bridged peptide androcin 10-1, and *Androctonus bicolor* (AC. no.: A0A0K0LCH4). (B) Down image shows the aa sequences alignment of meuAP-18-1 with homologues, including antimicrobial peptide meucin-18-1, Chinese M. eupeus (AC. no.: F6K5S5), antimicrobial peptide marcin-18, *Mesobuthus martensii* (AC. no.: F6K5S6), antimicrobial peptide marcin-18-1, *Mesobuthus martensii* (AC. no.: F6K5S7), antimicrobial peptide megicin-18, *Mesobuthus gibbosus* (AC. no.: A0A059U8Y9), antimicrobial peptide AMP2, *Odontobuthus doriae* (AC. no: A0A0U4LVY4), non-disulfide-bridged peptide androcin 18-1, *Androctonus bicolor* (AC. no.: A0A0K0LBT6), non-disulfide-bridged peptide androcin 18-4, *Androctonus bicolor* (AC. no.: A0A0K0LBT1), non-disulfide-bridged peptide androcin 18-2, *Androctonus bicolor* (AC. no.: A0A0K0LBT8). (C) Down image displys aa sequences alignment of meuPep34 with homologue peptide cellular protein AbCp-7 from *Androctonus bicolor* (Ac.no.: A0A0K0LC44). Conserved residues are represented in dark gray. Arginine and tryptophan are indicated with black color. Transmembrane domain is highlighted with yellow highlight. Residues with similar physicochemical properties are shown in light gray. Dash showes that there is not any residue in that position. The percentages of similarity with meuVAP-6, meuAP-18-1, and meuPep34 are brought in the right of alignment. Cystein residues are indicated with red rectangule.

**Fig. 3:**
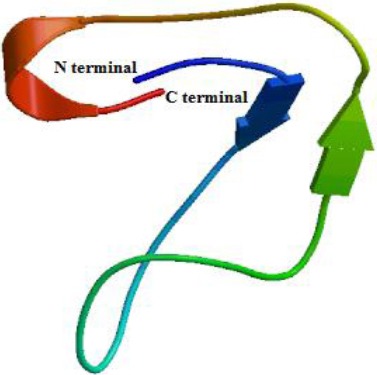
A three-dimensional model of meuPep34

The amino acid sequence alignments of meuVAP-6, meuAP-18-1, and meuPep34 with homologues in the UniProt database and the schematic image of peptides are indicated in Figures 2A, 2B, and 2C, respectively. The comparative sequence analysis of meuVAP-6 and meuAP-18-1 with other scorpion peptides revealed that both of them have some similarities with scorpion venom AMPs. Therefore, it can be concluded that meuVAP-6 and meuAP-18-1 are AMPs. The protein BLAST search and the alignment of meuPep34 with its counterparts indicated that meuPep34 is a peptide from scorpion as it is highly similar to AbCp-7 from scorpion *Androctonus bicolor*. The three-dimensional structure modeling of meuPep34 (Fig. 3) showed that meuPep34 has structural similarity with Bubble, a defensin protein, from *Penicillium brevicompactum*[10] with the coverage of 29% and the sequence DNA similarity of 41%. As a result, meuPep34 is supposed to be a new scorpion defensin-like peptide. In the predicted three-dimensional model, meuPep34 has three β-sheet strands.

MeuVAP-6 and meuAP-18-1 have no cysteine residue; therefore, they are classified in non-disulphide-bridged antimicrobial peptides (NDBPs), while meuPep34 contains 8 cysteine residues in which the pattern of CXCX_7_CX_12_CX_7_CX_13_CX_4_CX_10_C was found.

Predicted secondary structures and some physiochemical properties of meuVAP-6, meuAP-18-1, and meuPep34 are summarized in Table 1. Based on the positive net charges and the existence of basic amino acids (arginine and tryptophan [RW]) among the amino acids of all the three peptides (Fig. 2A, 2B, and 2C), we concluded that the described peptides are all entirely cationic.

**Table 1:** Predicted Secondary structure and some physiochemical properties of peptides described in this study

Peptide name	Secondary structure	Molecular weight	Isoelectric pI	Hidrophobicity index	Net charge at pH 7
meuVAP-6	MKSQTFFLLFLVVFLLAITQSEAFIGGVVSLLKNIFGKRSL RDMDTMKYLYDPSLSAADLKTLQKLMENY ccccceeehhhhhhhhhhccchhhhcchhhhhhcccccceeechhhhhhhccccc chhhhhhhhhhceec	8014.45	9.61	0.37	2.0
meuAP-18-1	MQWKKQLMVIFLAYFLVVNESEAFFGSLFKLATKIIPSLF RRKKERSIMKRDLENLFDPYQRNLEMDRLLKQLPNY cccccchhhhhhhhhhhcccchhhhhhhhhhhhhhchhhhhhhhhhhhhhhhhhcccc chhhhhhhhhhhhceeec	9289.96	10.42	-0.25	6.0
meuPep34	MKFSLFILLIVLTYVHYSLAGGCFCYSRTGTYCGFQREHH HLGGDCSRDNMYTCRGGNGALAGWEGRCRRGRCVGSN PAGRDYCPHGSERGRRKRFISNLEKI Cccceeeeeeeeeeeeeeccccceeecccccccceeeecccecccccccceeeceecc cccccccccccccceecccccccccccccccccchhhhhhceeec	11614.19	9.28	-0.47	8.9

The random coil is represented by “c”, the extended strand by “e” and the helix by “h”

## DISCUSSION

Scorpion venom gland is considered as a source of novel AMPs[3]. Considering the importance of AMPs as new anti-infective compounds, this study was designed to identify some novel anti-microbial peptides expressed in scorpion venom gland. In the current study, we presented three new cationic AMPs, including meuVAP-6, meuAP-18-1, and meuPep34.

Protein blasts of meuVAP-6 and meuAP-18-1 against homologues in the UniProt database indicates that N-terminal and C-terminal of both peptides are most highly conserved (Fig. 2a and 2B). Meucin-13 and Meucin-18-1 from Chinese *M. eupeus* with the identity of 91.4% and 92.1% are the most similar peptides to meuVAP-6 (Fig. 2A) and meuAP-18-1 (Fig. 2B), respectively. Regarding these similarities, it can be suggested that meuVAP-6 and meuAP-18-1 are the new variants of known peptides in the Chinese scorpion, or they are completly novel peptides. Moreover, the finding of identical peptides in the venom of different scorpion species has been indicated to be common[11]. Furthermore, the finding of similar peptides in scorpions from the same strain might be due to subspecies.

As mentioned above, meuVAP-6 and meuAP-18-1 are NDBPs. Zeng *et al*.[12] have classified the scorpion NDBPs into six subfamilies. Based on this classification, meuVAP-6 and meuAP-18-1 are clustered in third subfamily containing long chain peptides, including hadrurin, parabutoporin, BmKbpp, pandinin 1, opistoporin 1, and opistoporin 2. In NDBPs grouping of Harrison *et al*.[3], meuVAP-6 and meuAP-18-1 are also placed in the long-chain peptide group. Zeng *et al*.[12] have described some features for cDNA of NDBPs and their encoded precursors. These features are extended for meuVAP-6 and meuAP-18-1 and includes AAA or TTT motif at 3’UTR of cDNA (Fig. 1), the existence of a small neutral residue (glycine, alanine, or serine) at the cleavage site of signal peptide, the presence of one positively charged residue, Lys, in N-terminal region, as well as one negatively charged residue (glutamic acid or aspartic acid) at C-terminal region of each precursor (Fig. 2A and 2B). It has been claimed that the presence of a rich AAA or TTT motif at 3’UTR of cDNA have positive effect on the stability of RNA[12]. Although meuPep34 is not a NDBP, it is very rich in AAA or TTT motif at 3’UTR of its cDNA (Fig. 1), which may affect the RNA stability.

Based on the existence of signal peptide[13], it is suggested that all of the three described peptides are secretory peptides, i.e. when they are inserted into endoplasmic reticulum, the signal peptides are removed by a signal peptidase enzyme, and the mature peptide would be released in endoplasmic reticulum. Their transmembrane predicted domains (Fig. 2A, 2B, and 2C) consist of 23 amino acids and contain a large part of signal peptide plus four or six (six in meuVAP-6 and meuPep34, four in meuAP-18-1) beginning amino acids of mature peptides. It is proposed that the peptides attach to the membrane of endoplasmic reticulum through these domains.

It is known that the insertion of highly basic peptides into the cytosol perturb the integrity of the lipid bilayer[14]. On the other hand, the first and crucial step in the mode of AMPs action is interaction with microbial cells, which causes membrane perturbation. Disruption of the membrane, which is followed by some events such as cell wall biosynthesis or cell division, and/or translocation across the membrane to interact with cytoplasmic target, is leading to cell death[15]. It is assumed that the positively charged amino acids of AMP initially interact with the negatively charged lipid head groups of the outer surface of the cytoplasmic membrane, which leads to bringing AMP near to the cytoplasm membrane[15,16]. Therefore, it can be concluded that a peptide with more positive net charge could be more potent against the microbial cells. In addition to electrostatic interaction, hydrophobocity is another item affecting AMP activity[17,18]. After close proximity to a lipid bilayer by the electrostatic interactions, AMPs can interact with cell membrane through hydrophobic forces[19-21]. In a research investigating the activity and the properties of analogs of BmKn1 from the venom of scorpion *Buthus martensii Karsch*, it has been observed that higher AMP hydrophobicity can be translated into more antimicrobial activity[22]. The comparative analysis of the present study described AMPs based on estimated net charges (meuVAP-6<meuAP-18-1<meuPep34), and hydrophobicity index (meuVAP-6>meuAP-18-1>meuPep34) implies that the most positively charged peptide has less hydrophobicity index. However, hydrophobic interactions in comparison to electrostatic forces are not long-ranged[23], and it is suggested that the meuPep34 has more and the meuVAP-has less AMP activity.

A class of AMPs consisting of MeuVAP-6 has just 2R, while meuPep34 has 13R and 1W, and meuAP-18-1 has 6R and 1W. In a study by Liu *et al*.[24], the effect of chain length on the antimicrobial activity of RW-rich AMPs was evaluated with synthesis of AMPs series with difference in the size of RW repeat. The results showed that the length of RW has a linear relationship with antimicrobial activity, i.e. the antimicrobial activity will increase with the elevation of RW length. Therefore, this result can be considered as a further evidence for the antimicrobial potency of meuPep34. Other study revealed that extracellular matrix are not able to affect biofilms[25] although some antimicrobial peptides, which exert their function through the formation of diffusion barrier of RW-rich AMPs, can inhibit the biofilm growth of *E. coli* in a certain dose[26]. Hou *et al*.[27] demonstrated that the chain length of RW-rich AMPs is important for the inhibition of bacterial growth in *E. coli* biofilm since AMPs with more RW are more potent. This fact improves the possibility that meuPep34 having 13R and 1W could be the potent inhibitor of biofilms. Moreover, as it can be conferred from the amino acid alignment (Fig. 2C), just one uncharacterized scorpion homologue exists for meuPep34. However, based on structural modeling that indicated a similarity with a known defensin, we suggest that meuPep34 is possibly a defensin. The alignment of meuPep34 with its homologue revealed that unlike the meuPep34, its homologue has seven cysteine residues, which is one residue less than meuPep34. The difference in numbers of cysteines may affect the final structure of the peptide. In the present study, we have identified new peptides in the Iranian Scorpion Venom with putative antibacterial activity. Therefore, the result of this study may introduce novel antimicrobial peptides to pharmaceutical industry.
